# Best practices of judicial governance: A scoping review protocol

**DOI:** 10.1371/journal.pone.0329904

**Published:** 2025-08-28

**Authors:** Leandra Vilela Rodrigues Chaves, Marcos de Moraes Sousa, Woska Pires da Costa, Jéssica Traguetto, Flávio Manoel Coelho Borges Cardoso, Miguel de Matos-Torres

**Affiliations:** 1 Business, Accounting and Economics Faculty, Universidade Federal de Goiás, Goiânia, Goiás, Brazil; 2 Instituto Federal Goiano, Campus Rio Verde, Rio Verde, Goiás, Brazil; 3 Instituto Federal Goiano, Campus Morrinhos, Morrinhos, Goiás, Brazil; 4 Instituto Federal Goiano, Campus Ceres, Ceres, Goiás, Brazil; 5 Kent Business School, University of Kent, Canterbury, Kent, United Kingdom; Universitas Airlangga, INDONESIA

## Abstract

**Background:**

Enhancing performance in the public sector is closely tied to institutional structures, governance models, and the behavior of public officials. In the Judiciary, these factors significantly affect the effectiveness of court administration and justice delivery. Judicial governance is a complex and evolving concept encompassing standards and practices related to accountability, independence, resource management, and institutional performance, progressively integrating principles from public management reforms. Despite its growing relevance, the field remains fragmented, with limited evidence connecting international standards to best governance practices in judicial administration.

**Objective:**

This protocol outlines a scoping review designed to identify, map, and synthesize evidence on best practices in judicial governance, examining their relationship with the effective administration of justice and identifying research gaps to propose a future research agenda.

**Method:**

This review will follow the JBI methodology and the PRISMA-ScR guidelines. A comprehensive search will be conducted in databases such as Scopus, Web of Science, DOAJ, and JSTOR, as well as additional searches in grey literature. The PCC (Population, Concept, and Context) framework guided the eligibility criteria, and the PRESS 2015 checklist was used to validate the search strategy. The PRISMA-S checklist will inform the reporting of the search process. Studies of all designs and publication statuses will be considered, with no restrictions on language or publication date. Two reviewers will independently screen using Rayyan software, with a third reviewer resolving any disagreements. Data extraction will occur at two levels: general source information and specific content related to the review scope. Qualitative data will be analyzed using NVivo software, enabling categorization, descriptive synthesis, gap identification, and the development of a research agenda.

**Discussion:**

This scoping review aims to generate key evidence that can inform institutional standards and best governance practices to support evidence-based policymaking; while it does not assess the risk of bias, its systematic methodology and inclusion of grey literature enhance its relevance for future research and innovations in the justice sector. Through this scoping review, key evidence will generate insights that can enhance institutional standards and best practices in governance, enabling evidence-based policymaking. Although the review does not assess the risk of bias, its systematic approach and inclusion of grey literature strengthen its potential to support future research and governance innovations in the justice sector.

**Trial registration:**

OSF Registries, Jan 21, 2024: https://doi.org/10.17605/osf.io/agv3b.

## 1. Introduction

Performance improvement in the public sector is a critical organizational concern, intrinsically linked to the motivation and behavior of agents, institutional configurations, and the governance model adopted. These factors directly affect the effectiveness or ineffectiveness of management practices in solving public problems. The central perspective of contemporary public governance is the change in relations between society and the central government, representing a new paradigm still under construction and leading to new management formats intended to support the needs of increasingly demanding and constantly changing social groups [1].

In public organizations, adopting governance models is important for identifying the specific and distinct institutional arrangements configured in the public sector and enabling effective action strategies to increase service performance [2]. Good governance is ultimately associated with improved state performance and greater societal trust [3]. Within this broader movement, traditionally shielded from pressures related to efficiency and transparency, the Judiciary has increasingly been called upon to adopt governance principles similar to those implemented in other areas of the public sector. In Western democracies, this represents a paradigmatic shift, as the Judiciary assumes a central role alongside the legislative and executive branches, reinforced by its structural independence [4].

Modern court management encompasses budget oversight, technology, infrastructure, human resources, and communications, with special attention given to interactions between judges and court staff [5]. Within this framework, judicial governance emerges as a complex, multidimensional concept encompassing accountability, accessibility, independence, resource allocation, institutional environment, and performance. It can be defined as the set of rules, actions, and decisions undertaken by public agents operating within the justice system [6]. It has been gradually reframed to reflect broader institutional and inter-organizational dynamics. Today, judicial governance involves multiple stakeholders—including judges, lawyers, politicians, and civil society actors—who play critical roles in regulating and supervising judicial performance [7]. In European democracies, judicial governance is frequently associated with administration, financing, and independence, varying according to whether oversight lies with the executive branch or independent institutions [8].

Within this context, court leadership plays a pivotal role. Beyond managing administrative tasks such as hiring, budgeting, and infrastructure, court leaders with discretionary powers over case assignments—common in some European countries—can significantly influence judges’ careers, workloads, and perceptions of impartiality [9,10]. Strong judicial governance practices foster transparency, accountability, and ethical behavior—conditions necessary for addressing complex social and institutional challenges [3,11]. They also build stakeholder trust and promote an efficient and independent judiciary, which is essential for a nation’s social and economic development [12].

Therefore, comprehending the elements associated with judicial performance, such as available resources, management structures, and psychological predictors, is key for the effective administration of justice [13]. Reforms inspired by the New Public Management (NPM) movement have played a central role in reshaping judicial institutions. In the Netherlands, the transition from an autonomous judiciary model to one influenced by NPM principles resulted in budget constraints, increased workloads, concerns about declining decision quality, and perceived threats to judicial autonomy [4]. In Australia, on the other hand, judicial self-governance has gained ground, with internal councils and boards increasingly managed by judges through legal, political, and managerial approaches [14]. In emerging countries such as Brazil, governance reforms promoted by the National Council of Justice (CNJ) have introduced performance targets and management innovations into judicial strategic planning [6]. These transformations underscore the need for theoretical and empirical inquiry into judicial governance, particularly concerning its standards and best practices.

Nevertheless, despite these reforms, the literature on judicial governance remains fragmented. Conceptualizations vary significantly across institutional and political settings. In Afghanistan, public management and governance challenges are closely linked to global indicators such as the rule of law, judicial independence, political stability, and respect for human rights, all of which are situated within the broader framework of local governmental activities [15]. In the United States, corporate scandals and economic crises have led to broad efforts to improve governance in public institutions, especially regarding the composition and performance of governing boards [2].

These global developments reflect the broader evolution of public governance, which now emphasizes dynamic interactions between society and the state. This transformation has redefined the state’s role from an absolute authority to a collaborative governance partner, promoting increased public participation in decision-making processes [16]. However, judicial independence is a cornerstone of the rule of law and democracy, and political contexts characterized by populism often employ strategies that undermine this autonomy, leading to a decline in public trust in the judiciary [17]. Given this scenario, this study is expected to make a significant contribution to both academic and managerial fields. The findings may advance theoretical understandings of judicial governance, identify standards and best practices, and provide practical insights to enhance work processes within the justice system.

The initial considerations highlight the need for more theoretical and empirical studies on judicial governance [6,14]. An exploratory search, such as those conducted using Prospero, PubMed, the Cochrane Database of Systematic Reviews, and JBI Evidence Synthesis, was undertaken. No current or in-progress scoping reviews or systematic reviews on the topic were identified [18]. In light of this context, the present protocol outlines a scoping review to explore how governance in the public sector, particularly within the Judiciary, is constructed, applied, and evaluated. Specifically, it seeks to identify and synthesize international standards and best practices in judicial governance and analyze their relationship with the effective administration of justice.

The underutilization of scoping reviews in applied social sciences, in the field of judicial governance and public management, further underscores the relevance of this effort [7,14]. While some studies have attempted to map this area, few have done so systematically. The literature remains fragmented, with persistent debates surrounding the competencies and roles of different actors and the organizational structures of judicial institutions. These discussions are further complicated by the interdisciplinary nature of the subject, which encompasses political science, law, public administration, and management [7,14].

Given this landscape, the proposed scoping review aims to make meaningful contributions to academic and institutional domains. Its findings may help consolidate theoretical frameworks on judicial governance, identify globally accepted standards and best practices, and provide actionable insights to enhance the effectiveness and integrity of judicial institutions. Therefore, understanding the context in which justice is administered is of great interest, as judicial governance is a multidimensional concept encompassing accountability, accessibility, independence, resources and structures, governance practices, institutional environment, and performance [10]. Thus, judicial governance extends beyond the internal functioning of courts, relating to how institutions are managed and exert control over conflicts in the administration of justice and society as a whole. Effective judicial governance is essential for economic and social development and instrumental in fostering respect for the law, encouraging ethical conduct, and strengthening democratic institutions [3].

### 1.1. Study’s Objectives

Therefore, the primary objective of this study protocol is to outline the scoping review method, which aims to identify, map, and synthesize evidence illustrating the relationship between international models and best governance practices in the administration of justice. Thus, to accomplish the study’s goal, this scoping review study will focus on the following:

(1) International standards of judicial governance.
(2) Best practices of judicial governance.
(3) The relationship between standards and best governance practices in justice administration.
(4) Research gaps and limitations to propose a research agenda.

## 2. Methods

The scoping review has become a recognized scientific method that comprehensively maps a given topic from multiple perspectives, particularly in contexts where the quality assessment of the included studies is not the primary focus. In this sense, the aim is to identify key concepts, categorize research results, and identify gaps. Unlike systematic reviews, scoping reviews are advantageous for answering broad and exploratory research questions, as they encompass all sources, regardless of their quality. In this context, the purpose is to identify key concepts and categorize research findings. Scoping reviews are especially valuable for exploring new areas of investigation, clarifying essential concepts, or identifying research gaps [19]. The results of such studies and other evidence types may be presented as standalone findings or serve as a foundation for developing a subsequent systematic literature review [19].

A scoping review protocol was developed using the methodological framework [19] and consolidated methods for scoping reviews [20]. Thus, this protocol adheres to the recommendations established for this type of study [18,21–26]. Accordingly, the development of this protocol was guided by best practice recommendations from the JBI Scoping Review Methodology Group [20], and it will be reported following the Preferred Reporting Items for Systematic Reviews and Meta-Analyses extension for Scoping Reviews (PRISMA-ScR) [27] (see Supplementary Information S1 File).

### 2.1. Trial registration

This protocol has been preregistered on the Open Science Framework^®^ (OSF^®^ Registries, available at: https://doi.org/10.17605/osf.io/agv3b) [20,28,29] and published in this scientific journal prior to data extraction and the initiation of the review, to promote transparency [30,31] and ensure the rigor of this study. Any changes made to the protocol during the study will be updated in the trial registration and reported in the final manuscript presenting the results of the scoping review.

### 2.2. Information sources

The evidence sources include studies with quantitative, qualitative, and mixed methods designs, which will be considered [31]. Primarily, these sources were indexed in scientific databases and will be selected. All types of study designs, including reviews, meta-analyses, and other types of reports, will be included [24,31]. For systematic searches, a pre-established search string will be employed to identify studies aligned with the focus of this scoping review. Furthermore, the research team will adapt the search strategy for each selected database, incorporating all identified keywords and indexing terms [30,31]. The primary search will include the following databases: Scopus^™^, Web of Science Core Collection^™^, Directory of Open Access Journals (DOAJ), and Journal Storage (JSTOR^®^). These databases were selected based on identifying relevant studies in prior research on models and governance practices in the justice context.

Second, the references of the included studies will be screened recursively to identify additional potentially eligible studies [30,31]. No quality restrictions will be applied to provide the broadest possible overview of the field. Additionally, both published and gray literature will be considered, including government reports and documents from organizations available online. The search will include specific sources, such as Google Scholar^™^, Bielefeld Academic Search Engine (BASE), and Directory of Open Access Repositories (OpenDOAR), to expand the results as much as possible.

Neither primary nor secondary sources will be restricted by language or publication date [30–32]. On the other hand, dissertations, theses, and undergraduate monographs will be excluded. Finally, in each database, to maintain alignment with the search strategy, available filters will be used to exclude studies unrelated to the study’s objective, which is limited to the field of applied social sciences.

### 2.3. Search strategy

The key concepts in the research question and the inclusion criteria established for the study were considered to define the search strategy for this protocol. Some adjustments were made to the syntax of each database to optimize the search and allow for greater precision using advanced search tools. Searches were conducted using the titles, abstracts, and keywords from the metadata of the scientific databases [32].

The mnemonic “PCC” (Population/Participants, Concept, and Context) will be used to predetermine eligibility criteria and guide the identification of relevant studies [20], as it encompasses the most significant elements of the research focus [26]. The PCC framework is detailed in Table 1.

**Table 1:** PCC framework.

Component	Definition
P (Population/Participants)	Judiciary and judicial organizations are integral to public administration, responsible for delivering essential services to society [21]. In modern democracies, the judiciary protects the constitution and ensures the guarantee of human rights [22].
C (Concept)	The concept will consider evidence sources that explore the core concepts related to international standards and best practices of judicial governance.
C (Context)	The context, or setting, will consider evidence sources from any judicial system worldwide, including rules, strategies, resources, and institutional arrangements.

PCC is a strategy to aid in scoping the review, which defines the key elements of the research, i.e., ‘P’ to delineate the Population/Participants, ‘C’ to specify the Concept, and another ‘C’ to detail the Context.

To construct the search string, synonymous and similar terms were considered, as well as British and American English variations, singular and plural forms, and other term-specific nuances. To ensure the retrieval of all relevant studies, indexed terms and their synonyms or similar terms were grouped using the Boolean operator “OR” and, subsequently, these blocks were then combined using the “AND” operator [32]. The search will be performed using the terms specified in the strings (Table 2).

**Table 2:** Strings defined for the primary scientific databases search.

Blocks (PCC)	Keywords used
**#1** (Population/Participant)	“international standard” OR “international model” OR “international framework” OR “international guideline” OR “global standard” OR “international recommendation” OR “transnational standard” OR “international legal standard” OR “global framework”
**#2** (Concept)	“governance practice” OR “governance model” OR “governance framework” OR “governance standard” OR “governance strategy” OR “governance approach” OR “governance pattern” OR “governance procedure” OR “governance method” OR “governance perspective” OR “governance reference” OR “governance success model” OR “governance excellence” OR “best governance practice” OR “judicial governance” OR “justice governance” OR “court governance” OR “institutional governance” OR “effective governance” OR “high-quality governance” OR “judicial leadership”
**#3** (Context)	judiciary OR “justice system” OR “justice sector” OR “justice governance” OR “judicial system” OR “judicial organization” OR “judicial organisation” OR “judicial government” OR “judicial management” OR “judicial administration” OR “judicial oversight” OR “court organization” OR “court organisation” OR “court government” OR “court administration” OR “court management” OR “board administration” OR “board management” OR “tribunal administration” OR “tribunal management” OR “administration technique” OR “administration practice”
**Search string:**	**(#1) AND (#2) AND (#3)**

PCC is a strategy to aid in scoping the review, which defines the key elements of the research, i.e., ‘P’ to delineate the Population/Participants, ‘C’ to specify the Concept, and another ‘C’ to detail the Context.

The search strategy was validated using the evidence-based checklist from the Peer Review of Electronic Search Strategies (PRESS 2015) guideline [33]. The syntax was subsequently adapted to meet the specific requirements of each database, thereby broadening the scope and capturing relevant studies. Based on the systematic search string, data extraction will be conducted on a unique day using metadata files generated directly from the database platforms. The entire extraction process will be documented following the PRISMA for Search (PRISMA-S) guidelines [34] (see Supplementary Information S2 File). Finally, exhaustive searches in secondary data sources may occur at various moments; as these are not part of a systematic process, detailed documentation may not be feasible.

### 2.4. Eligibility criteria

We will include published and unpublished studies and evidence from scientific journals, with no restrictions, as long as they meet the following eligibility criteria. Evidence may be excluded if it meets at least one of the predefined exclusion criteria [32], as detailed below:

Inclusion criteria:

(i1) Evidence from studies retrieved from scientific databases using the strategy previously defined with a primary systematic search string or from various sources identified in the gray literature through exhaustive searches of secondary platforms.
(i2) Evidence that addresses international standards or best practices in judicial governance, or that explores the relationship between such standards and effective governance practices in the administration of justice.

Exclusion criteria:

(e1) Duplicates: If multiple articles have been published by the same author, on the same dataset, and on the same topic, only the most comprehensive among them will be considered. Duplicates will be removed following Bramer’s method [35], and we will conduct a manual review to confirm their exclusion [36,37].
(e2) Dissertations, theses, or undergraduate monographs.
(e3) Studies that are not fully available in the searched databases and cannot be accessed even after attempts to contact the authors have been made [24,37,38].
(e4) Articles written in a restricted language that cannot be appropriately translated [39]. This criterion will apply only after exhausting all translation options—international collaborators, artificial intelligence tools, and specialized services—and will be reported in the review findings [32].

### 2.5. Screening process

**Fig 1:**
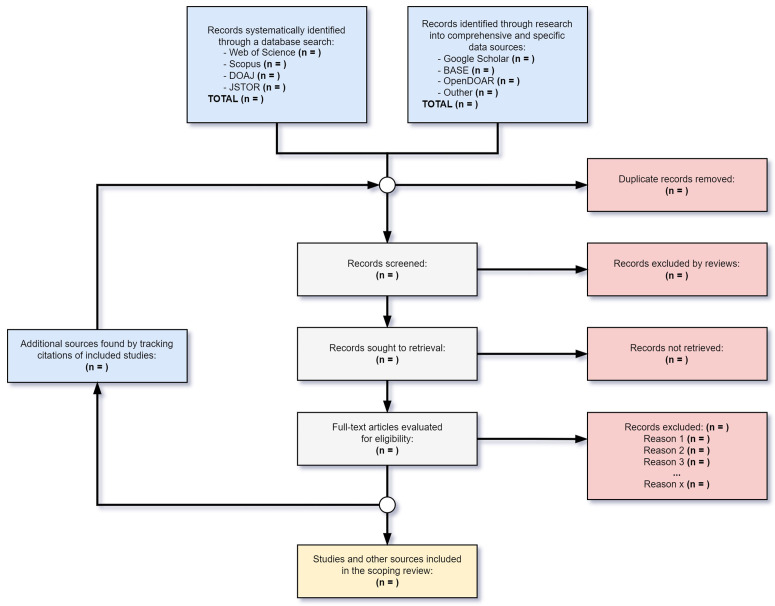
Scoping review flow diagram.

The screening process outlined in this protocol presents a priori description of the planned methodological steps for conducting the scoping review. All stages of the review process are illustrated in the flow diagram proposed for this type of study (Fig 1), which will guide reviewers from the selection of information sources through all intermediate stages—including the recursive citation tracking step mentioned earlier—up to the inclusion of studies and relevant information in the scoping review. The research questions will guide decisions and, most importantly, the eligibility criteria. Further, all findings from this process will be documented for inclusion in the final manuscript presenting the scoping review results.

Before the screening process, once the metadata from the scientific databases have been imported into the Rayyan^®^ software (Rayyan Systems Inc., Cambridge, MA, USA), the main reviewer will execute the deduplication process [40]. This online tool (https://www.rayyan.ai/) was designed to help conduct systematic reviews [41]. Thus, this software will perform all stages of the scoping review, with the reviewers’ blinding feature activated for evaluation [39].

In the first stage of screening, studies extracted from primary sources will be reviewed independently by two reviewers to ensure alignment with the inclusion criteria, based on the title and abstract of each text [42–44]. The study co-authors will act as reviewers, and if necessary, new reviewers may be added to ensure that the study remains manageable and that the scoping review remains feasible [42]. Any potential discrepancies will be resolved by a third independent senior reviewer [29,32]. In terms of secondary sources, the entire process will be conducted by the first author, who will serve as the main reviewer for this scoping review. In cases of uncertainty, a senior reviewer may be consulted to support decision-making.

In the second stage, the reviewers will read articles marked for full-text review to ensure that they meet the inclusion criteria and do not fall under any exclusion criteria [43,44]. Any discrepancies will be reviewed by an additional team member, who will decide on the article’s inclusion [43]. If a study is unavailable, one reviewer will contact the corresponding author to request the full text [29] and, if necessary, the related dataset.

Finally, all decisions made for each study will be duly labeled to ensure precise, transparent, and complete documentation of the entire process, including both inclusion and exclusion criteria, at the first and second stages of the review.

### 2.6. Review training

A pilot assessment will be conducted to train the reviewers involved in this study under the supervision of an experienced researcher. This training session aims to standardize the screening decisions based on the pre-established eligibility criteria [45] for this scoping review. It will also include guidance on using the Rayyan^®^ software, ensuring reviewers become familiar with its features and functionalities. This training will promote a standardized analysis process and help reviewers correctly label the studies at each review stage.

### 2.7. Data extraction

All data extracted from the evidence sources will be entered into a summarized table that includes information such as the study, source, objective, participants, context, concept, method, results, and other relevant findings (see Supplementary Information S3 File). This table will be reviewed and adjusted as necessary during the data extraction process [24,44,46]. The full extracted evidence will be compiled as supplementary material and made available alongside the scoping review publication as ‘Evaluated Data’.

Data will be extracted at two levels to support the objective of identifying, mapping, and synthesizing evidence related [24] to international standards and best governance practices in the administration of justice: i) general information about the source of evidence will be collected (e.g., author, year of publication, country of origin, and any reported conflicts of interest); ii) specific information relevant to the scoping review focus will be extracted, such as the nature of the standard, practice, or governance model addressed, the methodological approach employed, the type of data or context in which it was applied, the relationships drawn between standards and best practices, and whether key stakeholders were considered in the development or implementation of the evidence. This two-tiered data extraction method enables a comprehensive mapping of the existing landscape, supporting the identification of research gaps and informing future directions.

### 2.8. Data analysis

The data will be inductively coded in NVivo^®^ (QSR International Pty Ltd., Melbourne, VIC, Australia) across different approaches [42,47], capturing the range of methodological frameworks in the corpus. The results will be narratively synthesized, offering concise analyses of each strategy’s characteristics, strengths, and limitations. The study further seeks to determine whether these frameworks address gender inequity as a structural and systemic issue embedded in societal relations, rather than treating gender mainstreaming and equity merely as technical procedures. This approach facilitates a critical assessment of the interplay between theoretical perspectives, practical implementation, and the perpetuation of power asymmetries.

Data analysis will consist of basic descriptive [31] and reflexive thematic analysis [48]. The six thematic analysis steps were followed [49]: i) familiarization with the data, through repeated reading, and annotation; ii) generation of initial codes, through intense open-coding of data to generate an initial coding frame based on thematic categories rooted in the data; iii) identification of themes, through a detailed review of the coding frame to sort codes into potential themes; iv) review of themes, through refinement of the developing themes; v) definition and refinement of themes, through exploration of relationships within and between codes, and revision of thematic definitions; and vi) writing of the study findings. The results will be presented in tabular and diagrammatic formats to align with the review objective. A narrative summary will accompany the tabulated and charted results, describing how the results relate to the review objective and questions.

### 2.9. Methodological quality and risk of bias assessment

This scoping review will not assess the methodological quality of the included studies or the risk of bias associated with them. Assessing the risk of bias is not a mandatory step in scoping reviews, since the focus is not on producing clinical recommendations or causal inferences, but on describing the extent, variety, and characteristics of the literature on a given topic [18,27]. Therefore, the inclusion of studies will be based solely on the previously defined eligibility criteria, without considering their individual quality.

### 2.10. Presentation of results

The study screening process will be described using narrative, tabular, and visual formats to demonstrate how the results align with the objectives and research questions of the review [31]. The elements of the PCC inclusion criteria will be considered to guide the best format for presenting the review results to the audience [18]. Thus, the results will be organized in a spreadsheet and included as supplementary material. If necessary, the findings will be categorized and subcategorized objectively and concisely [32]. Additionally, as appropriate, quantitative data may be transformed into themes or categories [50]. The evidence gathered may also be presented using figures, diagrams, or other visual elements that best illustrate the clustering of factors and emerging patterns and trends [51,52]. Presenting the results in a suitable and detailed format will allow the reviewers to identify gaps in the literature and map the available evidence [18].

### 2.11. Ethics and dissemination

Ethical approval is not typically required for scoping studies, as they generally analyze previously published studies or publicly available evidence (i.e., secondary data). The results and conclusions obtained will be published in a peer-reviewed journal, contributing to the advancement of the research area and supporting further investigation.

## 3. Discussion

This scoping review protocol is expected to synthesize key evidence on the relationship between international standards and best governance practices in the administration of justice. The review will map the judicial organizations and management practices referred to in the literature, as well as their relationships with the core elements and dimensions of governance. These connections may offer valuable insights for improving the effectiveness and performance of judicial organizations. The findings are expected to make a meaningful contribution to the field, particularly by supporting the development of future research within the judiciary context. Additionally, the results may serve as a valuable resource for researchers, public administrators, and policymakers involved in designing and implementing governance strategies in the justice sector.

### 3.1. Strengths and limitations

As with any scoping review, the study derived from this protocol may present certain limitations. Firstly, due to resource constraints, including time and personnel availability, the selection of databases will be limited to a predefined set of scientific and gray literature sources. As a result, the search strategy may fail to retrieve some relevant studies, even if they are indexed in other databases. Secondly, based on the methodological characteristics of scoping reviews, this study will not assess the methodological quality or risk of bias of the included evidence. This characteristic may lead to the inclusion of studies with varying levels of rigor and reliability. Nevertheless, this decision is supported by the exploratory nature of scoping reviews, whose primary purpose is to map the existing evidence base comprehensively rather than appraise the quality of individual studies. Furthermore, the fact that we will not assess the quality of the sources may lead to the inclusion of studies with certain design deficiencies [46].

On the other hand, this study has strengths, such as the use of consolidated software to support the entire scoping review process, the search in several scientific databases—including multidisciplinary and comprehensive ones—as well as the use of alternative sources of gray literature and other types of evidence that could be integrated, generating solid and robust results. In addition, we propose a new diagram for conducting scoping reviews, incorporating recursive input by tracking citations of the included evidence. Finally, we describe our protocol in detail, with the intention of contributing to other researchers who wish to carry out studies similar to the one we propose.

## Supporting information

S1 File PRISMA for scoping reviews (PRISMA-ScR) checklist. (PDF)

S2 File Details of boolean search string used for each database. (PDF)

S3 File Data extraction summary form. (PDF)
