# Supplementary material for: Best practices of judicial governance: A scoping review protocol
Source: PLoS One. 2025 Aug 28;20(8):e0329904. doi: 10.1371/journal.pone.0329904 (PMC12393731; doi:10.1371/journal.pone.0329904)
Supplement: S1 File — (PDF) [file pone.0329904.s001.pdf]

Supplementary material (S1 File). The PRISMA-SrC checklist adapted to the scoping review protocol

Section/topic	Item nº	Checklist item	Information reported		Page
			Yes	No	
**TITLE**					
Identification	1	Identify the report as a 'scoping review protocol'.	✓		1
**ABSTRACT**					
Structured summary	2	Provide a structured summary that includes (as applicable): background, objectives, eligibility criteria, sources of evidence, charting methods, results, and conclusions that relate to the review questions and objectives.	✓		1
**INTRODUCTION**					
Rationale	3	Describe the rationale for the review in the context of what is already known. Explain why the review questions/objectives lend themselves to a scoping review approach.	✓		2–4
Objectives	4	Provide an explicit statement of the questions and objectives being addressed with reference to their key elements (e.g., population or participants, concepts, and context) or other relevant key elements used to conceptualize the review questions and/or objectives, preferably using the PCC framework.	✓		4 and 5
**METHODS**					
Protocol and registration	5	Indicate whether a review protocol exists; state if and where it can be accessed (e.g., a Web address); and if available, provide registration information, including the registration number.	✓		5
Eligibility criteria	6	Specify characteristics of the sources of evidence that will be used as eligibility criteria (e.g., years considered, language, and publication status), and provide a rationale, preferably based on the PCC framework.	✓		8 and 9
Information sources*	7	Describe all information sources in the search (e.g., databases with dates of coverage and contact with authors to identify additional sources), as well as the date the most recent search will be executed.	✓		6
Search	8	Present the full electronic search strategy for at least 1 database, including any limits that will be used, such that it could be repeated.	✓		6–8, and Supplementary Information S2
Selection of sources of evidence**	9	State the process for selecting sources of evidence (i.e., screening and eligibility) will be included in the scoping review.	✓		9–11

Section/topic	Item nº	Checklist item	Information reported		Page
			Yes	No	
Data charting process***	10	Describe the methods of charting data from the included sources of evidence (e.g., calibrated forms or forms that have been tested by the team before their use, and whether data charting was done independently or in duplicate) and any processes for obtaining and confirming data from investigators.	✓		9–12
Data items	11	List and define all variables for which data were sought and any assumptions and simplifications made.	✓		11–12
Critical appraisal of individual sources of evidence****	12	If done, provide a rationale for conducting a critical appraisal that includes sources of evidence; describe the methods used and how this information will be used in any data synthesis (if appropriate).		✓	NA
Synthesis of results	13	Describe the methods of handling and summarizing the data that will be charted.	✓		13
**DISCUSSION**					
Summary of evidence	14	Summarize the main results (including an overview of concepts, themes, and types of evidence available), link to the review questions and objectives, and consider the relevance to key groups.		✓	NA
Limitations	15	Discuss the limitations of the scoping review process.	✓		13–14
Funding	16	Describe sources of funding for the included sources of evidence, as well as sources of funding for the scoping review. Describe the role of the funders of the scoping review.	✓		15

Notes: JBI stands for the Joanna Briggs Institute; PRISMA-ScR stands for the Preferred Reporting Items for Systematic Reviews and Meta-Analyses extension for Scoping Reviews; and PCC is an abbreviation for Population, Concept, and Context. * Where sources of evidence (see second footnote) are compiled from, such as bibliographic databases, social media platforms, and Web sites. ** A more inclusive/heterogeneous term used to account for the different types of evidence or data sources (e.g., quantitative and/or qualitative research, expert opinion, and policy documents) that may be eligible in a scoping review as opposed to only studies. This is not to be confused with information sources (see first footnote). *** The frameworks by Arksey and O'Malley (6) and Levac and colleagues (7) and the JBI guidance (4, 5) refer to the process of data extraction in a scoping review as data charting. **** The process of systematically examining research evidence to assess its validity, results, and relevance before using it to inform a decision. This term is used for items 12 and 14 instead of "risk of bias" (which is more applicable to systematic reviews of interventions) to include and acknowledge the various sources of evidence that may be used in a scoping review (e.g., quantitative and/or qualitative research, expert opinion, and policy document). If any information is not reported or not evaluated, use the abbreviation 'NA' (not applicable) in the column entitled Page.
