# Supplementary material for: Best practices of judicial governance: A scoping review protocol
Source: PLoS One. 2025 Aug 28;20(8):e0329904. doi: 10.1371/journal.pone.0329904 (PMC12393731; doi:10.1371/journal.pone.0329904)
Supplement: S2 File — (PDF) [file pone.0329904.s002.pdf]

**Supplementary material (S2 File).** Details of Boolean search string used for each database.

Scopus™ via native interface

| Blocks and Returns | Search strings |
|---|---|
| #1<br>(Return: ‹$n_1$›) | TITLE-ABS-KEY("international standard" OR "international model" OR "international framework" OR "international guideline" OR "global standard" OR "international recommendation" OR "transnational standard" OR "international legal standard" OR "global framework") |
| #2<br>(Return: ‹$n_2$›) | TITLE-ABS-KEY("governance practice" OR "governance model" OR "governance framework" OR "governance standard" OR "governance strategy" OR "governance approach" OR "governance pattern" OR "governance procedure" OR "governance method" OR "governance perspective" OR "governance reference" OR "governance success model" OR "governance excellence" OR "best governance practice" OR "judicial governance" OR "justice governance" OR "court governance" OR "institutional governance" OR "effective governance" OR "high-quality governance" OR "judicial leadership") |
| #3<br>(Return: ‹$n_3$›) | TITLE-ABS-KEY(judiciary OR "justice system" OR "justice sector" OR "justice governance" OR "judicial system" OR "judicial organization" OR "judicial organisation" OR "judicial government" OR "judicial management" OR "judicial administration" OR "judicial oversight" OR "court organization" OR "court organisation" OR "court government" OR "court administration" OR "court management" OR "board administration" OR "board management" OR "tribunal administration" OR "tribunal management" OR "administration technique" OR "administration practice") |
| #4<br>(Return: ‹$n_4$›) | (#1) AND (#2) AND (#3) |
| | **Search refinement**:<br>⟨⟨Not applied⟩⟩<br>**Return after refinement**:<br>‹*N*› studies in the test carried out in ‹*date*›. |

Web of Science™ Core Collection via native interface

| **Blocks and Returns** | **Search strings** |
|---|---|
| #1 (Return: ‹$n_1$›) | TS=("international standard" OR "international standards" OR "international model" OR "international models" OR "international framework" OR "international frameworks" OR "international guideline" OR "international guidelines" OR "global standard" OR "global standards" OR "international recommendation" OR "international recommendations" OR "transnational standard" OR "transnational standards" OR "international legal standard" OR "international legal standards" OR "global framework" OR "global frameworks") |
| #2 (Return: ‹$n_2$›) | TS=("governance practice" OR "governance practices" OR "governance model" OR "governance models" OR "governance framework" OR "governance frameworks" OR "governance standard" OR "governance standards" OR "governance strategy" OR "governance strategies" OR "governance approach" OR "governance approaches" OR "governance pattern" OR "governance patterns" OR "governance procedure" OR "governance procedures" OR "governance method" OR "governance methods" OR "governance perspective" OR "governance perspectives" OR "governance reference" OR "governance references" OR "governance success model" OR "governance success models" OR "governance excellence" OR "best governance practice" OR "best governance practices" OR "judicial governance" OR "justice governance" OR "court governance" OR "institutional governance" OR "effective governance" OR "high-quality governance" OR "judicial leadership") |
| #3 (Return: ‹$n_3$›) | TS=(judiciary OR "justice system" OR "justice systems" OR "justice sector" OR "justice sectors" OR "justice governance" OR "judicial system" OR "judicial systems" OR "judicial organization" OR "judicial organizations" OR "judicial organisation" OR "judicial organisations" OR "judicial government" OR "judicial governments" OR "judicial management" OR "judicial administrations" OR "judicial administration" OR "judicial oversight" OR "court organization" OR "court organizations" "court organisation" OR "court organisations" OR "court government" OR "court governments" OR "court administration" OR "court administrations" OR "court management" OR "board administration" OR "board administrations" OR "board management" OR "tribunal administration" OR "tribunal administrations" OR "tribunal management" OR "administration technique" OR "administration techniques" OR "administration practice" OR "administration practices") |
| #4 (Return: ‹$n_4$›) | (#1) AND (#2) AND (#3) |
| | **Search refinement**:<br>⟨⟨Not applied⟩⟩<br>**Return after refinement**:<br>‹*N*› studies in the test carried out in ‹*date*›. |

Directory of Open Access Journals (DOAJ) via native interface

| **Blocks and Returns** | **Search strings** |
|---|---|
| #1<br>(Return: ‹$n_1$›) | ("international standard" OR "international standards" OR "international model" OR "international models" OR "international framework" OR "international frameworks" OR "international guideline" OR "international guidelines" OR "global standard" OR "global standards" OR "international recommendation" OR "international recommendations" OR "transnational standard" OR "transnational standards" OR "international legal standard" OR "international legal standards" OR "global framework" OR "global frameworks") |
| #2<br>(Return: ‹$n_2$›) | ("governance practice" OR "governance practices" OR "governance model" OR "governance models" OR "governance framework" OR "governance frameworks" OR "governance standard" OR "governance standards" OR "governance strategy" OR "governance strategies" OR "governance approach" OR "governance approaches" OR "governance pattern" OR "governance patterns" OR "governance procedure" OR "governance procedures" OR "governance method" OR "governance methods" OR "governance perspective" OR "governance perspectives" OR "governance reference" OR "governance references" OR "governance success model" OR "governance success models" OR "governance excellence" OR "best governance practice" OR "best governance practices" OR "judicial governance" OR "justice governance" OR "court governance" OR "institutional governance" OR "effective governance" OR "high-quality governance" OR "judicial leadership") |
| #3<br>(Return: ‹$n_3$›) | (judiciary OR "justice system" OR "justice systems" OR "justice sector" OR "justice sectors" OR "justice governance" OR "judicial system" OR "judicial systems" OR "judicial organization" OR "judicial organizations" OR "judicial organisation" OR "judicial organisations" OR "judicial government" OR "judicial governments" OR "judicial management" OR "judicial administrations" OR "judicial administration" OR "judicial oversight" OR "court organization" OR "court organizations" "court organisation" OR "court organisations" OR "court government" OR "court governments" OR "court administration" OR "court administrations" OR "court management" OR "board administration" OR "board administrations" OR "board management" OR "tribunal administration" OR "tribunal administrations" OR "tribunal management" OR "administration technique" OR "administration techniques" OR "administration practice" OR "administration practices") |
| #4<br>(Return: ‹$n_4$›) | (#1) AND (#2) AND (#3) |
| | **Search refinement**:<br>⟨⟨Not applied⟩⟩<br>**Return after refinement**:<br>‹*N*› studies in the test carried out in ‹*date*›. |

| Journal Storage (JSTOR®) via native interface | |
|---|---|
| **Blocks and Returns** | **Search strings** |
| #1 (Return: ‹$n_1$›) | ("international standard" OR "international standards" OR "international model" OR "international models" OR "international framework" OR "international frameworks" OR "international guideline" OR "international guidelines" OR "global standard" OR "global standards" OR "international recommendation" OR "international recommendations" OR "transnational standard" OR "transnational standards" OR "international legal standard" OR "international legal standards" OR "global framework" OR "global frameworks") |
| #2 (Return: ‹$n_2$›) | ("governance practice" OR "governance practices" OR "governance model" OR "governance models" OR "governance framework" OR "governance frameworks" OR "governance standard" OR "governance standards" OR "governance strategy" OR "governance strategies" OR "governance approach" OR "governance approaches" OR "governance pattern" OR "governance patterns" OR "governance procedure" OR "governance procedures" OR "governance method" OR "governance methods" OR "governance perspective" OR "governance perspectives" OR "governance reference" OR "governance references" OR "governance success model" OR "governance success models" OR "governance excellence" OR "best governance practice" OR "best governance practices" OR "judicial governance" OR "justice governance" OR "court governance" OR "institutional governance" OR "effective governance" OR "high-quality governance" OR "judicial leadership") |
| #3 (Return: ‹$n_3$›) | (judiciary OR "justice system" OR "justice systems" OR "justice sector" OR "justice sectors" OR "justice governance" OR "judicial system" OR "judicial systems" OR "judicial organization" OR "judicial organizations" OR "judicial organisation" OR "judicial organisations" OR "judicial government" OR "judicial governments" OR "judicial management" OR "judicial administrations" OR "judicial administration" OR "judicial oversight" OR "court organization" OR "court organizations" "court organisation" OR "court organisations" OR "court government" OR "court governments" OR "court administration" OR "court administrations" OR "court management" OR "board administration" OR "board administrations" OR "board management" OR "tribunal administration" OR "tribunal administrations" OR "tribunal management" OR "administration technique" OR "administration techniques" OR "administration practice" OR "administration practices") |
| #4 (Return: ‹$n_4$›) | (#1) AND (#2) AND (#3) |
| | **Search refinement**:<br>⟨⟨Not applied⟩⟩<br>**Return after refinement**:<br>‹*N*› studies in the test carried out in ‹*date*›. |
