# Supplementary material for: Best practices of judicial governance: A scoping review protocol
Source: PLoS One. 2025 Aug 28;20(8):e0329904. doi: 10.1371/journal.pone.0329904 (PMC12393731; doi:10.1371/journal.pone.0329904)
Supplement: S3 File — (PDF) [file pone.0329904.s003.pdf]

Supplementary material (S3 File). Data extraction summary form of the studies included in this scoping review.

(A) Citation detail	(B) Study location	(C) Study objective	(D) Population or Participant	(E) Concept	(F) Model or practice description	(G) Context	(H) Method	(I) Governance dimension	(J) Outcomes or Results	(K) Relationship between standard and practice	(L) Identified gaps	(M) Limitations	(N) Implications and/or recommendations
Smith & Lee (2018) *	European Union (EU)	To analyze how provincial courts have incorporated Council of Europe (CEPEJ) standards for judicial efficiency.	Magistrates and court administrators.	International judicial management standards promoted by the CEPEJ.	Application of the CEPEJ Checklist for the Quality of Judicial Systems in local courts.	Provincial civil courts in the EU judicial system.	Qualitative case study with documentary analysis and interviews.	Efficiency, transparency, performance management.	Partial adoption of the checklist, improvement in data collection, and creation of performance indicators.	The international standard served as the basis for judicial reform.	Lack of continuous training and standardization in the application of indicators.	Limited to three courts and a lack of longitudinal data.	Recommendation to institutionalize feedback mechanisms and expand training in judicial management.

Note: * Hypothetical example data.
